# Developing the medicinal plants sector in northern India: challenges and opportunities

**DOI:** 10.1186/1746-4269-2-32

**Published:** 2006-08-08

**Authors:** Chandra Prakash Kala, Pitamber Prasad Dhyani, Bikram Singh Sajwan

**Affiliations:** 1 National Medicinal Plants Board, Ministry of Health & FamilyWelfare, Government of India, 36- Janpath, Chandralok Building, NewDelhi- 110 001, India; 2 G.B. Pant Institute of Himalayan Environment & Development, Kosi-Katarmal, Almora, Uttaranchal- 263 643, India

## Abstract

The medicinal properties of plant species have made an outstanding contribution in the origin and evolution of many traditional herbal therapies. These traditional knowledge systems have started to disappear with the passage of time due to scarcity of written documents and relatively low income in these traditions. Over the past few years, however, the medicinal plants have regained a wide recognition due to an escalating faith in herbal medicine in view of its lesser side effects compared to allopathic medicine in addition the necessity of meeting the requirements of medicine for an increasing human population. Through the realization of the continuous erosion of traditional knowledge of plants used for medicine in the past and the renewed interest at the present time, a need existed to review this valuable knowledge of medicinal plants with the purpose of developing medicinal plants sectors across the different states in India. Our major objectives therefore were to explore the potential in medicinal plants resources, to understand the challenges and opportunities with the medicinal plants sector, and also to suggest recommendations based upon the present state of knowledge for the establishment and smooth functioning of the medicinal plants sector along with improving the living standards of the underprivileged communities. The review reveals that northern India harbors a rich diversity of valuable medicinal plants, and attempts are being made at different levels for sustainable utilization of this resource in order to develop the medicinal plants sector.

## Background

Forests have played key roles in the lives of people living in both mountains and lowland areas by supplying fresh water and oxygen as well as providing a diversity of valuable forest products for food and medicine [1]. The age-old traditional values attached with the various forest types and the varieties of forest products (i.e., medicinal plants) have gained tremendous importance in the present century [2,3]. Furthermore, the cosmetic industries are increasingly using natural ingredients in their products, and these natural ingredients include extracts of several medicinal plants [4]. India and China are two of the largest countries in Asia, which have the richest arrays of registered and relatively well-known medicinal plants [5]. Since the Indian subcontinent is well known for its diversity of forest products and the age-old healthcare traditions, there is an urgent need to establish these traditional values in both the national and international perspectives realizing the ongoing developmental trends in traditional knowledge. Apart from health care, medicinal plants are mainly the alternate income-generating source of underprivileged communities [6,7]; therefore, strengthening this sector may benefit and improve the living standard of poor people. A great deal of traditional knowledge of the use of various plant species is still intact with the indigenous people, and this fact is especially relevant with the mountainous areas such as the Himalaya due to less accessibility of terrain and comparatively slow rate of development [8,9].

The ongoing growing recognition of medicinal plants is due to several reasons, including escalating faith in herbal medicine. Allopathic medicine may cure a wide range of diseases; however, its high prices and side-effects are causing many people to return to herbal medicines which have fewer side effects [10]. The instant rising demand of plant-based drugs is unfortunately creating heavy pressure on some selected high-value medicinal plant populations in the wild due to over-harvesting. Several of these medicinal plant species have slow growth rates, low population densities, and narrow geographic ranges [11,12]; therefore they are more prone to extinction [13]. Conversely, because information on the use of plant species for therapeutic purpose has been passed from one generation to the next through oral tradition, this knowledge of therapeutic plants has started to decline and become obsolete through the lack of recognition by younger generations as a result of a shift in attitude and ongoing socio-economic changes [8,14]. Furthermore, the indigenous knowledge on the use of lesser-known medicinal plants is also rapidly declining [10]. Through the realization of the continuous erosion in the traditional knowledge of many valuable plants for medicine in the past and the renewal interest currently, the need existed to review the valuable knowledge with the expectation of developing the medicinal plants sector.

The present paper is therefore concerned with the following major objectives: 1) discovering the role, value, diversity and potential in medicinal plants resources, 2) assessing various aspects of medicinal plant sector, which includes challenges and opportunities, and 3) suggesting recommendations based on existing information for the benefit and development of medicinal plants sector in northern India.

### Use and diversity in medicinal plants

In India, of the 17,000 species of higher plants, 7500 are known for medicinal uses [15]. This proportion of medicinal plants is the highest proportion of plants known for their medical purposes in any country of the world for the existing flora of that respective country (Table 1). Ayurveda, the oldest medical system in Indian sub-continent, has alone reported approximately 2000 medicinal plant species, followed by Siddha and Unani (Table 2). The Charak Samhita, an age-old written document on herbal therapy, reports on the production of 340 herbal drugs and their indigenous uses [16]. Currently, approximately 25% of drugs are derived from plants, and many others are synthetic analogues built on prototype compounds isolated from plant species in modern pharmacopoeia [17].

**Table 1:** Distribution of medicinal plants.

**Country or region**	**Total number of native species in flora**	**No of medicinal plant species reported**	**% of medicinal plants**	**Source**
World	297000	52885	10	Schippmann et al. 2002
India	17000	7500	44	Shiva 1996
Indian Himalayas	8000	1748	22	Samant et al. 1998

**Table 2:** The status of various medical systems in India.

**Characteristics**	**Medical Systems**				
	
	**Ayurveda**	**Siddha**	**Unani**	**Tibetan**	**Homeopathy**
Medicinal plants known	2000	1121	751	337	482
Licensed pharmacies	8533	384	462	-	613
Hospitals	753	276	74	-	223
Dispensaries	15193	444	1193	-	5634
Registered practitioners	438721	17560	43578	-	217460
Under graduate college	219	6	37	-	178
Post graduate college	57	3	8	-	31

Modified after Anonymous 2004, 2005 [79,80]

The northern part of India harbours a great diversity of medicinal plants because of the majestic Himalayan range. So far about 8000 species of angiosperms, 44 species of gymnosperms and 600 species of pteridophytes have been reported in the Indian Himalaya [18], of these 1748 species are known as medicinal plants [19]. The maximum medicinal plants (1717 species) have been reported around the 1800 m elevation range. On the regional scale, the maximum species of medicinal plants have been reported from Uttaranchal [3], followed by Sikkim and North Bengal [19]. The trans-Himalaya sustains about 337 species of medicinal plants [8], which is low compared to other areas of the Himalaya due to the distinct geography and ecological marginal conditions [20].

Several plant species are endemic to the Himalayan region. Out of total known number of higher plants from India, approximately 46% are endemic to the Himalaya [21]. Of the total medicinal plant species, sixty-two species of medicinal plants are endemic to the Himalaya and 208 extend their distribution to the adjacent areas, and are therefore classified as near endemic [21]. Over 200 species of Himalayan medicinal plants are consumed raw, roasted, boiled, fried, cooked, or they are used in the form of oil, spices, jams or pickles [19,22]. The indigenous communities use some medicinal plant species as a source of food, fodder, timber as well as various other ethnobotanical purposes. For example, apart from the use of *Myrica esculenta *and *Terminalia bellirica *as medicines, the fruits of these species are edible, the leaves are used for fodder and the wood is used for fuel [22]. Approximately 81 species of Himalayan medicinal plants are known to be used for the extraction of oil. Of the total 675 species of Himalayan wild edibles, 171 are used for the treatment of diseases [23]. The crop plants diversity is also a source of traditional medicine [24].

Apart from the human use, animal husbandry uses many plant species as its primary source of healthcare in northern India [25,26]. The reliance on medicinal plants is also due to cultural preferences [27,28]. Medicinal plants have strong acceptance in religious activities of north Indian native communities, who worship the plants in the form of various gods, goddesses and minor deities [29,30]. *Origanum vulgare, Saussurea obvallata, Ocimum sanctum, Cedrus deodara, Cynodon dactylon, Aegle marmelos, Juniperus communis, Musa paradissica, Nardostachys grandiflora, Zanthoxylum armatum, Ficus benghalensis*, and *Ficus religiosa *are examples of the medicinal plants highly used for medicinal as well as a religious purposes by the Hindus in northern India. The Buddhist community in northern India regards *Terminalia chebula *as an important medicine as well as sacred fruit. It has been stated long ago that the therapeutic potency of medicinal plants is more effective and better suited to a person of a particular region or culture in which the plant is naturally growing [31]. This idea has given a way to the development of a new drug for heart patients of specific ethnic groups in African countries [32].

### Challenges in medicinal plants sector

The continuous increase in human population is one of the causes for concern in meeting the daily requirements of food and medicine as the economy and livelihoods of human societies living in developing countries primarily depend on forest products. This phenomenon is leading to continuous erosion of forest and the forest products [25], thus making challenge to meet the requirements as well as to conserve useful bio-resources. More and more species are being gradually added in the Materia Medica; however, the standards of their purity and correct identification do not keep pace with the process of expansion [33]. The market prices for medicinal plants and derived materials provide only a limited insight into the workings of the market, and not on the precise information of profits, supply and demand. We have identified the following major features and challenges on the basis of examining the existing knowledge on the medicinal plants sector.

#### Rising demand

**Figure 1:**
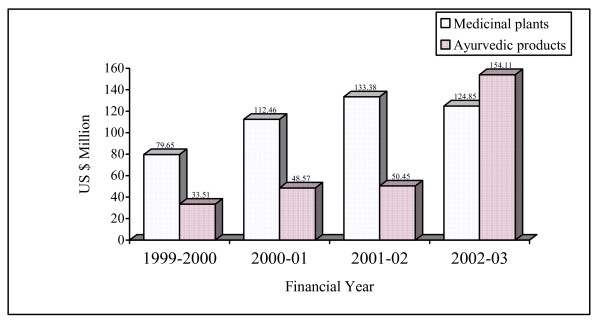
Annual export trends in medicinal plants and Ayurvedic products of India over past four financial years {Source: Prahalathan 2004 [74]}.

The World Health Organization (WHO) has estimated the present demand for medicinal plants is approximately US $14 billion per year [34]. The demand for medicinal plant-based raw materials is growing at the rate of 15 to 25% annually, and according to an estimate of WHO, the demand for medicinal plants is likely to increase more than US $5 trillion in 2050. In India, the medicinal plant-related trade is estimated to be approximately US $1 billion per year [35]. According to an estimate, the quantity of export of Ayurvedic products produced in India has tripled between last two financial years (2001–2002 and 2002–2003; Figure 1).

The projected escalating demand of medicinal plants has led to the over-harvesting of many plants from wild, which subsequently results in the loss of their existing populations. For example, the large quantity of Himalayan yew (*Taxus baccata*) has been gathered from the wild since its extract, taxol, was established as a use in the treatment of ovarian cancer. *Aconitum heterophyllum, Nardostachys grandiflora, Dactylorhiza hatagirea, Polygonatum verticillatum, Gloriosa superba, Arnebia benthamii *and *Megacarpoea polyandra *are other examples of north Indian medicinal plant species which have been overexploited for therapeutic uses and have subsequently been placed today in rare and endangered categories. Many medicinal plant species are used in curing more than one disease [36,37], and as a result, these species are under pressure due to over collection from wild. For example, *Hemidesmus indicus *is used to cure 34 types of diseases; *Aegle marmelos *31, *Phyllanthus emblica *29, and *Gloriosa superba *28 (Table 3). Over-exploitation and continuous depletion of medicinal plants have not only affected their supply and loss of genetic diversity, but have seriously affected the livelihoods of indigenous people living in the forest margins [17].

**Table 3:** Important medicinal plants in short supply and prioritized for the research and development.

**Sl. No.**	**Botanical name**	**Hindi name**	**Part used**	**No. of uses^a^**	**Important uses**	**Quantity required tonnes/annum^b^**	**Species in short supply^c^**	**Species prioritized for R & D^d^**
1	*Acacia catechu *(L. f.) Willd.	Khair	Root, bark	16	Asthma, bronchitis	2.4	√	-
2	Aconitum ferox Wall.	Vatsnabh	Root	6	Rheumatism	-	-	√
3	*Aconitum heterophyllum *Wall.	Atees	Root	12	Fever, cough, piles, stomachache	0.55	√	√
4	*Aegle marmelos *(L.) Correa	Bel	Fruit, bark	31	Dysentery, diarrhea, fever	-	-	√
5	*Alpinia galanga *(L.) Willd.	Kulanjan	Bulb	2	Health tonic	0.22	√	-
6	*Andrographis paniculata *(Burm. f.) Wall.	Kalmegh	Whole plant	22	Malaria, liver complaints, blood purifier	-	-	√
7	*Aquillaria malaccensis *Lamk.	Agaru	Leaf, resin	2	To remove fish spine from throat	0.17	√	-
8	*Artemisia maritima *L.	Kunja	Whole plant	7	Antiseptic, blood purifier	0.33	√	-
9	**-**	Ashtavarga	**-**	**-**	**-**	0.095	√	-
10	*Asparagus racemosus *Willd.	Shatavari	Root	22	Dysentery, cough, cut and wounds	-	-	√
11	*Bacopa monnieri *(L.) Penn.	Brahmi	Whole plant	20	Brain tonic, blood purifier, fever	-	-	√
12	*Berberis aristata *DC.	Kingora	Root, stem	4	Eye diseases	-	-	√
13	*Cassia angustifolia *Vahl	Senna	Root	3	Rheumatism	-	-	√
14	*Chlorophytum tuberosum *Bak.	Safed musli	Tuber	4	Leucorrhoea, sexual tonic	-	-	√
15	*Coleus barbatus *Benth.	Patharchur	Root	3	Tonic, blood pressure	-	-	√
16	*Commiphora wightii *(Arn.) Bhandari	Guggul	Resin, bark	9	Asthma, typhoid	2.3	√	√
17	*Crocus sativus *L.	Kesar	Flower	-	-	-	-	√
18	*Curculigo orchioides *Gaerten.	Kali musli	Root	26	Asthma, dysentery, tonic	2.25	√	-
19	*Curcuma zedoaria *(Christ) Rosc.	Kachora	Rhizome	4	Jaundice, blood pressure	0.225	√	-
20	*Embelia ribes *Burm. f.	Jheum	Seed, fruit	4	Skin diseases, leprosy	-	-	√
21	*Garcinia indica *Choisy	Kokam	Fruit	2	Skin diseases	-	-	√
22	*Gloriosa superba *L.	Kalihari	Rhizome	28	Snake bite, leprosy, tonic	-	-	√
23	Glycyrrhiza glabra	Mulethi	-	-	-	-	-	√
24	*Gymnema sylvestre *(Retz.) R. Br.	Gudmar	Root, leaf	8	Gastric disorders, eye diseases	-	-	√
25	*Hemidesmus indicus *(L.) Br.	Anantmul	Root	34	Cough, hypertension, dysentery	1.2	√	-
26	Mallotus philippensis Muell.-Arg.	Kamela	Fruit	16	Constipation, skin diseases, ulcer	0.155	√	-
27	*Myrica esculenta *Ham. ex Don	Kaphal	Bark, fruit	12	Asthma, fever, cough	0.225	√	-
28	Myristica fragrans Hoult.	Jaiphal	-	-	-	0.33	√	-
29	*Nardostachys jatamansi *(Don) DC.	Jatamansi	Rhizome	18	Bronchitis, blood purifier, hysteria	0.66	√	√
30	*Nelumbo nucifera *Gaertn.	Kamalphool	Fruit, seed	4	Cholera, diarrhea, tonic	0.31	√	-
31	*Ocimum sanctum *L.	Tulsi	Seed, leaf	18	Fever, vomiting, liver complaints, blood purifier	-	-	√
32	*Phyllanthus amarus *Schum & Thonn	Bhui amla	Whole	3	Jaundice, aphrodisiac, dysentery	-	-	√
33	Phyllanthus emblica L.	Amla	Fruit	29	Constipation, diabetes, tonic	-	-	√
34	*Picrorhiza kurrooa *Benth.	Katuki	Rhizome	13	Headache, fever, dysentery, anemia, asthma	1.55	√	√
35	*Piper cubeba *L. f.	Kabab chini	Fruit	7	Cholera, fever, cough	0.335	√	-
36	*Piper longum *L.	Pippal	Root	16	Indigestion, child birth, dysentery	-	-	√
37	*Pistacia chinensis *Bunge	Kakadshingi	Fruit	3	Scorpion bite, dysentery	0.45	√	
38	*Plantago major *L.	Isabgol	Whole	7	Wounds, weakness, constipation	-	-	√
39	*Rauvolfia serpentina *(L.) Benth. ex Kurz.	Sarpagandha	Root	14	Malarial fever, snake bite	-	-	√
40	*Santalum album *L.	Chandan	Wood	7	Dysentery, skin diseases	-	-	√
41	*Saraca asoca *(Roxb.) De Wilde	Ashok	Bark, leaf	4	Heart disorder, tonic	6.8	√	√
42	Saussurea costus (Falc.) Lipsch.	Kut	Root	7	Dysentery, asthma, ulcer	0.43	√	√
43	*Smilex *sp	Chopchini	Root	12	Menstrual complaints, small pox	0.55	√	-
44	*Solanum nigrum *L.	Makoy	Fruit	27	Jaundice, piles, skin diseases	-	-	√
45	*Swertia chirayita *(Roxb. ex Flem.) Karsten.	Chirata	Whole plant	16	Malarial fever, blood purifier	2.5	√	√
46	*Tinospora cordifolia *(L.) Merr.	Giloe	Whole plant	22	Jaundice, tonic, bone fracture	-	-	√
47	*Valeriana jatamansi *Jones	Tagar	Root, leaf	4	Epilepsy, urinary complaints	0.275	√	-
48	*Withania somnifera *(L.) Dunal	Ashwagandha	Root, leaf	14	Eye complaints, asthma, cough	-	-	√
49	*Wrightia tinctoria *Br.	Indrajava	Bark, latex	14	Toothache, piles, dysentery	0.418	√	-

^a ^Based on Jain 1991 [81]

^b, c ^Medicinal plants in short supply and quantity required according to the Planning Commission, Government of India

^d ^Prioritized species of medicinal plants for research and development according to the National Medicinal Plants Board, Government of India

- Not known

More than 95% of the 400 plant species used in preparing medicine by various industries are harvested from wild populations in India [38]. Harvesting medicinal plants for commercial use, coupled with the destructive harvest of underground parts of slow reproducing, slow growing and habitat-specific species, are the crucial factors in meeting the goal of sustainability [39,40]. Harvesting shoots and leaves of medicinal plants may decline their photosynthetic capacity, and as well as the potential for survival and effective propagation. Medicinal plants tolerance to harvest varies with climatic conditions as the temperate herbs become highly vulnerable to harvest of individuals [41]. Furthermore, rising demand with shrinking habitats may lead to the local extinction of many medicinal plant species.

#### Increasing rarity

The continuous exploitation of several medicinal plant species from the wild [42] and substantial loss of their habitats during past 15 years [43] have resulted in population decline of many high value medicinal plant species over the years. The primary threats to medicinal plants are those that affect any kind of biodiversity used by humans [17,44]. The weakening of customary laws, which have regulated the use of natural resources, are among the causes of threatening the medicinal plant species [40,45]. These customary laws have often proved to be easily diluted by modern socio-economic forces [4]. There are many other potential causes of rarity in medicinal plant species, such as habitat specificity, narrow range of distribution, land use disturbances, introduction of non-natives, habitat alteration, climatic changes, heavy livestock grazing, explosion of human population, fragmentation and degradation of population, population bottleneck, and genetic drift [14,46-48]. Additionally, natural enemies (i.e., pathogens, herbivores, and seed predators) could substantially limit the abundance of rare medicinal plant species in any given area [49,50].

In addition to the consumption of medicinal plants by animals, there are physical ailments in humans, which are cured by different species of the same genera. For example, the malarial fever is treated by many species of *Swertia *(e.g. *Swertia chiraiyta, S. angustifolia*, and *S. cordata*). Similarly, different species of *Berberis *(e.g. *Berberis aristata, B. asiatica, B. lycium, B. chitria *and *B. jaeschkeana*) are used as a source of berberidine to cure certain eye diseases. Furthermore, different species of the same genera contain different proportions of chemical quantity, and there is a preference over their demand; however, the degree of threat for their exploitation is relatively lower than those species, which do not have alternatives

An estimated 4,000 to 10,000 species of medicinal plants face potential local, national, regional or global extinction, with subsequent serious consequences for livelihoods, economies and health care systems [51]. Although, a few studies exist on the rare and endangered medicinal plant species of the northern India [8,14,48,52,53], none of these studies have complete data set for even a single species. In 2003, 71 rare and endangered medicinal plant species have been assessed for the northwest Himalaya during the Conservation Assessment and Management Plan workshop, according to the guidelines of the World Conservation Union. In northern India, *Aconitum *is the rarest genus with 5 species, followed by *Rheum *with 4 rare species. Out of the 71 rare medicinal plants, 92% are in active trade; 74% are traded nationally and 35% are traded internationally [52].

The meager availability of data on the population and quantum of rare species in nature, however, has restricted their categorization to a few species on the basis of herbarium collection and by consultation by a few experts [14]. The present assessments are also questioned for their validity on the assignment of threat categories to the species, including the number of taxa in danger for specific area. The problems in assessing the species is increased in the mountainous region, especially high altitude areas because of tough and inaccessibility of the terrain, inhospitable climatic conditions, and short life cycle of plants. Most of the available data have been collected from the easily accessible areas in these mountains. Indigenous communities and commercial herb gatherers also raid these same areas for collection of medicinal plants. Therefore, the estimated population density of categorized rare medicinal plants is not precise because it differs the areas that never and hardly undergone any collection of such rare medicinal plant species [54].

#### Cultivation of medicinal plants

Information on the propagation of medicinal plants is available for less than 10% and agro-technology is available only for 1% of the total known plants globally [55,56]. This trend shows that developing agro-technology should be one of the thrust areas for research. Furthermore, in order to meet the escalating demand of medicinal plants, farming of these plant species is imperative. Apart from meeting the present demand, farming may conserve the wild genetic diversity of medicinal plants. Farming permits the production of uniform material, from which standardized products can be consistently obtained. Cultivation also permits better species identification, improved quality control, and increased prospects for genetic improvements. Selection of planting material for large-scale farming is also an important task. The planting material therefore should be of good quality, rich in active ingredients, pest- and disease-resistant and environmental tolerant. For the large scale farming, one has to find out whether monoculture is the right way to cultivate all medicinal plants or one has to promote polyculture model for better production of medicinal plants.

Studies conducted on the agro-forestry of medicinal plants elsewhere suggest that since many medicinal plant species prefer to grow under forest cover, agroforestry offers a convenient strategy for their cultivation as well as conservation through: 1) integrating shade tolerant medicinal plants as lower strata species in multistrata system, 2) cultivating short cycle medicinal plants as intercrops in existing stands of tree crops, 3) growing medicinal tree as shade providers and boundary markers, and 4) inter-planting medicinal plants with food crops [17]. Notwithstanding, it is understood that the cultivation of medicinal plants is not an easy task as the history of medicinal plants farming reflects. Many farmers in trans-Himalayan region of northern India have replaced the medicinal plants farming with common crops [i.e., peas (*Pisum sativum*), potatos (*Solanum tuberosum*) and hops (*Humulus lupulus*)] due to the lengthy cultivation cycle of medicinal plants like *Saussurea costus *[7]. The cost of many medicinal plants in northern India is lower than many seasonal vegetables [58], which is a cause of scanty farming of medicinal plants.

Attempts are being made by different organizations to cultivate various medicinal plant species, including rare and endangered categories. Agro-technology for about 20 species of rare and endangered medicinal plants of the northern India has been developed by different organizations [52]. However, the per hectare cost of cultivation, total annual production and cost benefit ratio fluctuate with different medicinal plant species. Out of 10 selected rare and endangered medicinal plant species, *Rheum emodi *was calculated as a most beneficial cash crop of the medicinal plant in terms of net income generation in northern India (Table 4). At present, however, the farming of most of the medicinal plant species is being operated on a small scale and is restricted to a few hectares of land in various states of northern India. There is an uncertainty of obtaining the necessary permits from government agencies for cultivation of medicinal plants. Additionally, many farmers are unaware about the agency responsible for issuing permits. If the farmers are not granted permits needed to cultivate, they are forced to sell their products on the illegal market, which exposes them to action by government agencies and the exploitation by middlemen [14,59].

**Table 4:** Seedling survival, total cost of cultivation, and net income by cultivation of 10 important species of the rare and endangered medicinal plants.

**Species**	**Seedling survival (%)**	**Seed required per hectare (gm)**	**Total cost of cultivation (US $)**	**Total production (kg/hectare)**	**Income (US $)**	**Net income (US $)**
*Aconitum balfourii*	60–70	614	2117	495	2692	575
*Aconitum heterophyllum*	60–70	614	2258	83	4528	2270
*Angelica glauca*	70–80	800	1630	1000	2174	544
*Carum carvi*	>90	500	1652	650	2826	1826
*Nardostachys jatamansi*	50–60	600	2723	1129	6135	1412
*Picrorhiza kurrooa*	50–60	64	1117	612	1663	546
*Podophyllum hexandrum*	50–60	32,125	2718	4000	5218	2500
*Rheum emodi*	60–70	600	3044	9880	17183	14140
*Rhem moorcroftianum*	50–60	600	3044	4100	7130	4087
*Saussurea costus*	80–90	500	1783	3500	2283	500

Source: Nautiyal and Nautiyal 2004 [82].

#### Bio-prospecting and bio-piracy

The former remote green forests have now become part of a dynamic, profit-seeking economy and demanding pluralistic politics worldwide. Reducing the pressure on various forest products, especially on the medicinal plants, is therefore a tough challenge both for policy makers and for economists. Medicinal plants are the local heritage with global importance. The Convention on Biological Diversity at Rio had agreed on a framework that would help the biodiversity to be utilized in a prudent and controlled way. Bio-prospecting, at present, occurs in an environment of suspicions and growing tensions between the bio-piracy and rights of sharing benefits between the developing and developed countries [60]. Most of the issues relating to the protection of the legal status for indigenous knowledge and compensation of the indigenous herbal practitioners for that knowledge are extremely complicated. There are arguments for the present state of compensation or benefit sharing under the intellectual property rights, which is being considered a new legal form of bio-piracy by one group, whereas other groups argue that the intellectual property right is a legal tool to protect the rights of knowledge holders [61].

Different ways and systems for awarding patents on the medicinal plants in India, United States, Europe, Canada and other countries have widened the confusion [62]. In many countries, the plants and inventions directed to the plants and the plant products (i.e., seeds, flowers, gums, and resins) are not eligible for filing a patent. In United States, however, any living organism derived by human invention, such as by breeding or by laboratory-based manipulation, can be filed for awarding patent. The Indian Protection of Plant Varieties and Farmers Rights Act of 2001 recognizes the contribution of farmers who actively participate in the breeding programs. Furthermore, this act contains provisions for benefit sharing whereby local communities are acknowledged as a contributor of plants [62].

Unfortunately, there is a wide gap between developed and developing nations such as India on patenting the products. For example, out of the 3,125,603 patents filed in 91 countries, only 301,177 or 9.6% are registered in developing countries while the rest is in industrialized countries. Of these, only 0.2% of the total and 2.3% of those registered in developing countries belong to residents. In addition, 97.7% of the total patents filed thus far are in the name of non-residents, who apply solely to control export markets in developing countries [63]. Developing nations and many scientists who want to exploit medicinal plants demand more specific rules about the recording of nationality of samples and sharing of their benefits between the nations of origin, the inventor, and the commercial sponsors. Some developed nations are not inclined to accept such provisions. These conflicts have frustrated many scientists who believe that natural products remain the most promising source for new drugs. To mitigate such conflicts and gear up to find out new sources for drugs, the representatives of 188 nations at Kuala Lumpur Conference in 2004 agreed to try to build such a framework that would be acceptable to all signatories and thus the proposed framework will be placed for consideration at the next meeting in 2006 going to be held in Brazil [60].

#### Strengthening legalized market system

Besides government agencies, there are numbers of stakeholders ranging from herb gatherers, local middlemen, urban traders, wholesalers, manufacturers, exporters and herbal healers in the medicinal plants trade sector. The marketing system in medicinal plants sector is largely unregulated and inequitable [4]. The medicinal plant collectors are generally the marginal farmers and laborers. They get cash income to meet their basic requirements for food, health and children education by selling medicinal plants [42]. They are often unaware about the real market prices of many medicinal plant species. Generally, in medicinal plants sector, there is a top down approach and even the many stakeholders at the bottom are not aware of the rising demand of their product and the availability of its market. In some villages of Chamoli district of Uttaranchal, the farmers had cultivated Kut (*Saussurea costus*) and Dolu (*Rheum emodi*) but they were unable to sell them due to lack of knowledge on the marketing system. Conversely, many medicinal plant species are traded through illegal channels [42].

The other constraints in the medicinal plants sector are: 1) slow rate of production of many medicinal plants, 2) long gestation period, 3) shortage of suitable cultivation technology, 4) production of small quantity, 5) unscientific harvesting, 6) paucity of research on the high yielding varieties, 7) inefficient processing techniques, 8) fluctuation in demand and supply, 9) poor quality control procedures, 10) scarcity of good manufacturers, (11) poor marketing infrastructure, and 12) poor coordination among different stakeholders [1,3,8,14,51,59,64]. On many occasions, the wild medicinal plants are preferred by manufactures compared to the cultivated ones, as there is a general feeling that wild plant species contain better chemical contents. The variation in chemical contents also depends upon the harvesting seasons of species and different stages of species growth. The medicinal plant sector is largely less documented and inadequately regulated. The economy generated by this sector is therefore, not precise because of the enormous illegal trade. The economic benefits and management cost for wild populations are poorly estimated on several occasions [4,65].

### Opportunities in developing the medicinal plants sector

For developing the 'herbal industries', the northern India possesses a rich diversity of medicinal plant species across the various forest types along an altitudinal gradient (as discussed in the use and diversity of medicinal plants). Such a high diversity of medicinal plants would be helpful for further scientific research on exploring their medical efficacy, value addition, and use in curing various old and new diseases [3]. India has already established a reputation as a low-cost manufacturer of high quality generic drugs in the global market [66]. This fact can be used as an important tool for the marketing of herbal products produced in India. It is expected that India's aim to build a golden triangle between traditional medicine, modern medicine, and modern science will be a boon for developing the traditional herbal medicine and the medicinal plants sector [66].

#### Existing policies

In the National Five Year Plans of India, the medicinal plants sector has been identified as an integral part of the Indian System of Medicine and Homeopathy [67]. In 1983, the National Health Policy recognized that the large stock of health manpower in Ayurveda, Siddha, Unani, Homeopathy and Naturopathy had not been adequately utilized; therefore, steps need to be taken to move towards a meaningful integration of the indigenous and modern systems of medicine [68]. The Planning Commission and the National Medicinal Plants Board (NMPB) of the Government of India have prepared a policy document on the promotional and commercial aspects of the medicinal plants sector. In addition, the NMPB has prioritized 32 and Planning Commission has enlisted 24 medicinal plant species for research and development in order to meet the desired aim of the medicinal plant sector (Table 3). The Biodiversity Act 2002 has framed many rules for sustainable utilization of medicinal plants and to mitigate the chances of bio-piracy [69].

According to Uttaranchal state action plan, the Forest Department of the state will have to identify two major areas in each Forest Division; namely the conservation area and the developmental area. The conservation areas will be selected based on their rich medicinal plants diversity and marked for *in-situ *conservation and complete protection in the concerned Forest Division. In the developmental areas, apart from protection of the existing bio-resources, the medicinal plant species of the neighboring areas will also be introduced and cultivated at a large scale. The remaining areas in the Forest Division will remain open for sustainable harvesting of the medicinal plant species. A Joint Harvesting Team, composed of medicinal plants experts, Forest Department officials and some selected local people, will be constituted, which will decide the extent of annual harvesting of the desired medicinal plant species [70]. The various policies at national and state level and their subsequent implementation will provide an opportunity in the advancement of medicinal plants sector. This model of conservation and cultivation of the medicinal plants may be useful for generating the raw material for the 'Herbal Industries' as well as for ensuring the conservation of the rare medicinal plants.

#### Institutional support

In India, many government and non-government organizations have had the focused attention on improving the medicinal plants sector (Table 5). Opportunities for funding have been created to assist the person who is willing to work and to build capacity of the medicinal plants sector. According to the mandate of NMPB, the projects may be submitted for funding within two major schemes: *viz*., a promotional scheme and a commercial scheme. The major thrust areas within the promotional scheme are: 1) survey and inventory of medicinal plants, 2) *in-situ *conservation and *ex-situ *cultivation of selected medicinal plants, 3) production of quality planting material, 4) diffusion of knowledge through education and communication, 5) promotion of global and domestic market system, and 6) strengthening research, development and man power. Within the commercial scheme, the major thrust areas are: 1) bulk production of medicinal plants and ensuring supply of quality planting material, 2) expansion of selected medicinal plants farming areas, 3) value addition in harvesting, processing and marketing of medicinal plants, and 4) developing innovative marketing mechanism.

**Table 5:** Major institutions involved in funding projects to the medicinal plants research in India.

**Institutions**	**Funding for major areas in medicinal plants research**
National Medicinal Plants Board, NMPB	Survey, documentation, cultivation, marketing, conservation
Department of Science & Technology, DST	Taxonomy, ecology, pathology, survey, propagation, documentation, cultivation, conservation
Council for Scientific & Industrial Research, CSIR	Ecology, taxonomy, biochemistry, survey, documentation, cultivation, genetics, agro-technology, conservation
Indian Council of Medical Research, ICMR	Breeding, value addition
All India Council for Technical Education, AICTE	Management technology
Department of Biotechnology, DBT	Agro-technology, molecular biology, biochemistry, rural bio-technology
Defense Research & Development Organization, DRDO	Agro-technology, survey, documentation, conservation
Indian Council of Agricultural Research, ICAR	Breeding, pathology, molecular biology
Ministry of Environment & Forest, MoEF	Survey, documentation, conservation, management, ecological impact assessment, cultivation
National Bank for Agriculture and Rural Development, NABARD	Cultivation, marketing
University Grant Commission, UGC	Ecology, biochemistry, survey, documentation
Herbal Research and Development Institute, HRDI	Survey, documentation, nursery development
G.B. Pant Institute of Himalayan Environment & Development, GBPIHED	Survey, documentation, cultivation, conservation

Apart from the two major themes, the role of NMPB is to co-ordinate with the different ministries, departments, organizations, state and union territory Governments in order to develop and strengthen the medicinal plant sector. One of the major roles of NMPB is to make contacts with national and international organizations devoted to similar mandates and goals. A total of 35 State Medicinal Plants Boards have also been created by NMPB for the smooth functioning of the medicinal plants sector. Approximately 35,000 hectares of land has been selected and brought under cultivation under the supervision of NMPB for the large-scale farming of commercially important medicinal plants. One of the schemes of NMPB is a contractual farming in which any group, institution or person that possesses at least 3 years of experience in medicinal plants sector would be eligible to receive at least 30% financial assistance of the total project cost [71].

Ten years before establishment of NMPB (during 1992–93 financial years), a single project was launched to study the medicinal plants of the Himalayan region [72]. During past one decade, there has been a considerable expansion in the Himalayan medicinal plants research as it is evident that many projects have been launched exclusively on the medicinal plants during that period. Of the total 4254 projects sanctioned by NMPB over past 5 years, 732 projects have been sanctioned to 11 hill states in northern India. All these projects are expected to strengthen the medicinal plants sector, to bridge the gaps and to meet the challenges in developing the sector. In addition to major funding organizations, the G.B. Pant Institute of Himalayan Environment and Development has sanctioned projects on the medicinal plants of northern India under the scheme of Ministry of Environment & Forests (Table 6). The Council of Scientific and Industrial Research is building up a Traditional Knowledge Digital Library, which will contain > 35,000 herbal medical formulations used in Ayurvedic system of medicine [69].

**Table 6:** Status of project sanctioned on medicinal plants research and other disciplines by G.B. Pant Institute of Himalayan Environment and Development under the Integrated Eco-development Research Programs during 1992–2004

**Serial number**	**States (In Indian Himalaya)**	**Total project sanctioned on various disciplines**	**Number of Projects sanctioned on medicinal plants**	**% of projects sanctioned on medicinal plants by states**
1	Uttaranchal	88 (49)	12 (44)	14
2	Himachal Pradesh	24 (13)	3 (11)	12
3	Jammu-Kashmir	12 (6)	5 (18)	42
4	Sikkim	4 (2)	-	-
5	Manipur	6 (3)	-	-
6	Assam	21 (12)	3 (11)	14
7	Arunachal Pradesh	10 (5)	1 (4)	10
8	Meghalaya	8 (4)	2 (7)	25
9	West Bengal (Hills)	3 (2)	1 (4)	33
10	Mizoram	1 (0.5)	-	-
11	Nagaland	3 (2)	-	-
12	Tripura	-	-	-

*Total*		**180**	**27**	**15**

Values in parentheses are in percentage

The National Bank for Agriculture and Rural Development (NABARD) has established a policy with a viewpoint to develop a suitable environment for financial institutions by providing bankable models for some 50 medicinal and aromatic crops with the unit cost and scale of finance at the state level. Additionally, NABARD assists in supporting the capacity building of prospective entrepreneurs through Rural Entrepreneurs Development Programs by providing 100% refinancing [73,74]. NABARD has also close linkages with the NMPB and different State Medicinal Plants Boards. Attempts have been made by various organizations at different levels to establish and promote the medicinal plants sector.

### Recommendations for developing the medicinal plants sector

The present worldwide interest in plant-based medicines of Indian origin needs to be harnessed by reframing a clear policy for the promotion of commercial cultivation, research and development, and for the increase in exports of medicinal plants. For the development of the medicinal plant sector, there is a need to develop the coordinated efforts at each stage (e.g. research, cultivation, collection, storage, processing, manufacturing and marketing), which would be supported by an appropriate policy framework. Some problems and their remedies for the medicinal plants-based economic venture identified in this review are given in Table 7.

**Table 7:** Assessment of problems and remedies for medicinal plant based economic venture in the northern India

**Activity**	**Problems**	**Possible remedy**
Cultural system	Adoption of traditional medicinal knowledge on preparing herbal medical formulations is declining through generations.	Incentives should be given to the traditional herbal healers for preparation of herbal formulations, and attempts should be made to organize them.
	Traditional knowledge on many less known medicinal plant species has declined rapidly.	Documentation of such less known medicinal plant species should be made without any further delay.
Collection	Continued illegal collection from wild has led to depletion of many important species.	Enforcement of existing Acts (e.g. Wildlife Protection Act, Forest Act, Biodiversity Act etc.).
	Mostly collected and processed by un-trained persons.	Training should be given for collection and processing.
	Competition for over-stocking has led to over-harvesting.	Large-scale farming of medicinal plants should be promoted.
Cultivation	Agro-technology is not available for many valuable medicinal plant species. Development of agro-technology is mainly focused on the low productive and high cost rare and endangered medicinal plant species.	Development of agro-technology and promotion of rural bio-technology for large scale cultivation of economically important species also. Farmers should be encouraged by providing incentives, training and awareness on the latest developments and policies related to the medicinal plants. Selection of planting material for cultivation should be based on their habitats, locality, climate and elevations.
	High risk in farming, long gestation period, and low prices of medicinal plants discourage farmers to cultivate medicinal plants.	Introduction of mixed cropping system to reduce the risk
	Issuing license or permit to farmers for growing medicinal plants is a time consuming process, and farmers are sometime not aware of the process.	Process of issuing permits for cultivation of medicinal plants should be made easier and faster.
	Small and scattered land holdings of the farmers, and cultivation is restricted to small plots near the farmer's houses.	Restoration of barren lands and allocation of land at one place based on farmer's choice and consensus.
	Unavailability or low availability of irrigation facility	Rain water harvesting and construction of check dams on rivers and rivulets for irrigation purposes
	Lacking of linkages among different stakeholders.	Development of capacity building programs for all stakeholders.
Role of Biotechnology	Low success rate in developing planting materials.	Need of in-depth research to enhance the rate of success.
	Low yield unable to meet the commercial needs.	Development of high yielding varieties.
Marketing	The supply chain of medicinal plants is quite large and primary producers are dependent on the middlemen and still they face difficulty in selling the product.	Direct selling to industry by producers should be encouraged. Buy-back arrangements between farmers and pharmaceutical companies might be useful.
	Improper sharing of benefits due to lack of awareness among farmers and herb collectors on the real prices of medicinal plant.	Need of diffusion of information by distribution of pamphlets and conducting awareness programs on various aspects of medicinal plants.
	Lacking of well-planned marketing infrastructure for medicinal plants.	Development of infrastructure with the help of various stakeholders including medicinal plants board.
Bio-prospecting	Low awareness on the values of resources and traditional knowledge.	Documentation of traditional knowledge on medicinal plants and their uses.
	The younger generations of herbal practitioners are not keen to adopt the tradition as a profession.	Renew the available herbal formulations by standardizing their efficacy, and to establish a Social Capital Trust for herbal practitioners in order to promote the tradition.
	Unequal distribution of profits to the low profile stakeholders such as farmers and herb gatherers.	Sharing of benefits should be on the basis of labor and efforts.
Conservation	Essential health commodity and maximum dependency on wild stock.	Setting up medicinal plants conservation areas.
	Encroachment by outsiders and illegal collection from wild.	Enforcement of Forest and Wildlife Protection (Acts).

Selection of medicinal plant species for cultivation is an initial important step for the development of the medicinal plants sector. Economic feasibility is the major rationale for a decision to bring medicinal plant species into cultivation. Apart from the priority species selected by the Planning Commission and the NMPB, the rare species banned for collection from the wild should also be taken on a priority basis for cultivation because a majority of such species are very expensive, have high demand and low supply. Cultivation may not be economical if a medicinal plant species is abundant in the wild and easily collected. Therefore, the less abundant species in the wild should be promoted for the large-scale cultivation. Farming of any medicinal plant species should be brought into practice only after the reliable cultivation technology of the concerned species is available. A large variation in climatic and soil conditions in northern India sustain a variety of medicinal plant species, which may be cultivated according to their niche.

For developing the medicinal plants sector, there is a need to: 1) document indigenous uses of medicinal plants, 2) certify raw material for quality control, 3) develop and improve the agro-technology for valuable medicinal plants, 4) officially recognize and protect the customary laws of indigenous people, 5) prepare a clear policy for granting permits for cultivation within stipulated time, 6) conduct regular research and training on better harvesting and processing techniques, 7) investigate various pathological agents infecting medicinal plants, 8) setup a community-based management of medicinal plants farming and marketing, 9) analyze the market policies, 10) monitor and evaluate the status of medicinal plants with the assistance of local communities, 11) conserve the critical habitats of rare medicinal plant species, and 12) share benefits judiciously arising from local people's knowledge on medicinal plants. These attempts may reduce dependency on wild resource base, and generate alternative income opportunities for the rural and underprivileged communities [4,48,75,76].

The medicinal plants sector can be improved if the agricultural support agencies would come forward to help strengthen the medicinal plants growers, and if research institutions would help the plant growers by improving their basic knowledge about cultivation practices [16]. Awareness and interest of farmers, supportive government policies, assured markets, profitable price levels, access to simple and appropriate agro-techniques, and availability of trained manpower are some of the key factors for successful medicinal plants cultivation [59]. The diffusion of any available scientific knowledge on medicinal plants should be made operational by a network structure of communication. Currently there are number of herbs which are used in curing diseases but are not documented in details due to a lack of communication and relatively low frequency of their uses. The traditional uses of low profile and lesser-known medicinal plants should also be documented to disseminate their therapeutic efficacy by preparing well acceptable medicines and also to reduce the pressure on over-exploited species.

Apart from the more than 9992 licensed pharmacies with 717319 registered practitioners of Ayurveda, Siddha, Unani and Homeopathy in India (Table 2), there are many unregistered herbal practitioners in India who prepare their own traditional herbal formulations for curing different diseases, and the available herbal formulations should be standardized for their efficacy. Such scientifically prepared medicine will place herbal medicine in a proper perspective and help in getting a broad global market. Some people do try to take advantage through the introduction of less effective medicinal plants in the name of well-established high-value medicinal plants. Such attempts must be discouraged. To discourage such malpractices, the certification of raw material should be done for quality control by using the modern tools such as thin layer chromatography and high performance thin layer chromatography. In addition, high performance liquid chromatography (HPLC), volumetric analysis, gas chromatography, column chromatography, and gravimetric determinations may be used for standardization and for quality control [35,77,78]. Various factors relating to pathological agents (e.g. bacteria, virus, fungi and nematodes) and infected medicinal plants should be investigated by identifying the various symptoms of diseases such as mildew, rust, necrosis, spots, blight, rot, wilt, galls, curl and canker to produce the healthy farming of medicinal plants.

On many occasions, the collection of planting material, especially of rare and endangered medicinal plant species from natural habitats for various experimental purposes by researchers, also poses a threat on their natural populations in wild. The researchers must be aware on the germination potential, seedlings and rhizomes survival strategies of the desired species collected from wild for scientific experiments. Researchers must plant a similar number of individuals back in nature after completion of research work on the collected species [50]. There is also a communication problem between researchers and farmers. This communication problem limits a researcher's capability to deal with the farmers' problems [4]. Hence, communication links between researchers, extension services of institutions, and farmers should be strengthened.

The majorities of current research programs on the medicinal plants conservation are being shifted from ecosystems to species levels. Although there are protected areas across the Himalaya, most of the protected areas have a focused attention on the preservation of faunal diversity except for a few protected areas such as the Valley of Flowers in North West Himalaya and the Tipi Orchid Sanctuary in North East Himalaya. There is not a single protected area focusing on the conservation of medicinal plants. Thus, there is an urgent need for identification and notification of areas for the conservation of medicinal plants on a priority basis. Most of the documentation and research on indigenous uses of medicinal plants is focused on the human aspect. Animal husbandry is the backbone of economy in a majority of indigenous societies. Maintaining the good health of livestock will benefit these indigenous societies. Since there are many medicinal plant species used in curing the various animal related disorders and diseases, the research work also needs to be done on the uses of medicinal plants for curing livestock diseases.

Being a part of tradition, there are many other social issues attached with medicinal plants sector. The indigenous knowledge on harvesting, storage and usage of medicinal plants built over centuries needs to be taken into account for improving the sector and allocating scare resources among the competing demands. Development of medicinal plants farming, encouragement of traditional herbal use and herbal healers, establishing medicinal plants conservation areas, establishing the Social Capital Trust for herbal healers, establishment of linkages among various stakeholders, etc., are among some of the social issues that need to be honored and addressed properly. The folklore on several medicinal plants and the formulation developed by using them is well recognized in different ethnic communities living in northern India. These folklores should be brought into laboratory for validation.

## Conclusion

The traditional medical systems of northern India such as Ayurveda and Tibetan are part of a time-tested culture and honored by people still today. These traditions have successfully set an example of natural resource use in curing many complex diseases for more than 3,000 years. Many advantages of such eco-friendly traditions exist. The plants used for various therapies are readily available, are easy to transport, and have a relatively long shelf life. The most important advantage of herbal medicine is the minimal side effects, and relatively low cost compared to the synthetic medicines. The success of medicinal plants sector mainly depends on the awareness and interest of the farmers as well as its other stakeholders, supportive government policies, availability of assured markets, profitable price levels, and assess to simple and appropriate agro-techniques. The successful establishments of medicinal plants sector may help in raising rural employment, boost commerce around the world, and contribute to the health of millions.
